# Comparison of Sexual Experience and Behavior between Bipolar Outpatients and Outpatients without Mood Disorders

**DOI:** 10.1155/2016/5839181

**Published:** 2016-04-17

**Authors:** Jennifer Downey, Richard C. Friedman, Elizabeth Haase, David Goldenberg, Robinette Bell, Sidney Edsall

**Affiliations:** ^1^Department of Psychiatry, New York State Psychiatric Institute and Columbia University College of Physicians and Surgeons, New York, NY 10032, USA; ^2^Department of Psychiatry, Weill Cornell Medical School, New York, NY 10021, USA; ^3^Department of Psychiatry, Carson Tahoe Health System, Carson City, NV 89701, USA; ^4^Department of Psychiatry, Weill Cornell Medical College, New York, NY 10021, USA; ^5^Department of Psychiatry, University of Colorado School of Medicine, Aurora, CO 80045, USA; ^6^Behavioral Health and Recovery Services, San Mateo County Psychiatric Department, San Mateo, CA 94403, USA

## Abstract

Sexual behavior over the past year of 32 outpatients with Bipolar disorder is compared to that of 44 Comparison patients that had never had an episode of affective illness. Subjects were outpatients treated with drugs and psychotherapy in routine office practice. Differences in sexual behavior between the two groups as a whole were minimal, but meaningful differences emerged when subgroups were compared. Compared to control men, Bipolar men had had more partners in the last year and were more likely to have had sex without condoms. Compared to Bipolar females, Bipolar males had more sex partners, had more sex with strangers, and were more likely to have engaged in homosexual behavior. Even so, some patients in the Comparison group also had engaged in risky sexual behavior. They had failed to use condoms and had had sex with strangers and prostitutes during the previous year.

## 1. Introduction

This paper discusses a study of sexuality in Bipolar outpatients treated in private practice in comparison to outpatients without affective disorder or schizophrenia.

The evidence that sexuality is often increased in adults with Bipolar disorder during hypomanic or manic episodes includes controlled and uncontrolled studies and clinical case reports [1]. The DSM-IV-TR description of mania listed hypersexuality in criteria B #6 and #7 as “increased goal driven behavior…sexually” and/or “excess involvement in pleasurable activities that have a high potential for painful consequences: for example, sexual indiscretions” [2]. The DSM-5 [3] had not been published at the time this study was carried out but continues to list hypersexuality as a frequently occurring feature of bipolar mania and hypomania.

The study we discuss below was designed to assess a dimension of sexuality not included in most research to date. We were interested in sexuality as part of the person's temperamental trait behavior, rather than simply being influenced by affective state. We sought to assess whether sexuality was different among individuals with Bipolar disorder even when they are well treated and stable compared with those without affective disorder. As part of the preparation for the study, our group reviewed the literature on sexuality in Bipolar disorder, which we now discuss.

*Controlled Studies of Sexuality in Patients with Bipolar Disorder*. We found 10 English-language controlled studies of sexuality in patients with Bipolar disorder. These were conducted in different subcultures over a time-span of over 45 years during which diagnostic criteria and clinical treatment changed dramatically. Most studies asked only a few questions about sex, and there was no uniformity across studies of the questions asked or instruments used to acquire information. Medication status of patients was sometimes not reported and the phase of illness studied ranged from mania to depression. Some studies found aspects of sexuality to be increased in Bipolar patients but some were negative.

*Positive Studies*. Clayton et al. [4] found increased sexuality in manic compared to depressed patients. Jamison et al. [5] found increased sexuality in manic or hypomanic patients compared to major depressive controls. Additionally, 40 percent of the Bipolar I and II patients in this study reported elevated sexuality as an enduring interepisodic trait. Raja and Azzoni [6] found that Bipolar patients were more likely to be having stable sexual relations, to be married, and to have more frequent intercourse than schizophrenic patients at hospital admission. Dell'Osso et al. [7] reported that increased interest in sex and frequent change of sexual partners were more common among patients with Bipolar disorder compared with patients with unipolar depression and normal controls. Mazza et al. [8] found that Bipolar I patients placed more value on sex, had more interest in sex, and desired and engaged in intercourse more frequently than Bipolar II or Comparison patients.

*Negative Studies*. Beigel et al. [9] reported higher lifetime incidence of periods of celibacy in Bipolar than unipolar patients. Spalt [10] reported that Bipolar patients were less sexually active than unipolar patients, or patients with affective disorders secondary to primary psychiatric or medical illness or nonaffective disorder patients. Casper et al. [11] studied depressed Bipolar patients and reported more loss of interest in sex among them compared to normal controls. Raboch [12] found that Bipolar inpatients in states of both mania and depression did not differ from gynecology patient controls in sexual knowledge and attitude, function, or developmental history. Additionally, Frank et al. [13] compared couples in which one partner was Bipolar to nonpsychiatrically disordered control couples and found no difference in satisfaction between them.

*Uncontrolled Studies of Sexuality in Patients with Bipolar Disorder*. The uncontrolled studies include Allison and Wilson [14] who studied sexual behavior in manic or hypomanic patients. In their group, of 13 women, seven had markedly increased sexual display and provocative or seductive behavior. The investigators did not comment on provocative or seductive behavior in the 12 men in the study. However, 7/12 men and 10/12 women experienced a marked increase in libido in an elevated mood state. Similarly, Winokur et al. [15] found that 65 percent of manic episodes were characterized by increased sexuality, evenly divided between those in committed and noncommitted relationships. Carlson and Goodwin [16] studied 20 unmedicated manic patients admitted for four months for an observational study of a complete episode and found hypersexuality in 80 percent, although no specific sexual behavior is described. Loudon et al. [17], however, found sexual preoccupation in only 25 percent of 16 manic inpatients who started medication by the third day of admission.

In a retrospective chart review study of 1,000 Bipolar II patients, Akiskal [18] found that 40 percent of cyclothymic patients had “episodic or unexplained promiscuity or extramarital affairs” and that Bipolar II patients manifested diverse types of sexual excess including sexual infidelity, overt bisexuality, and sexual activity many times per day.

Of the uncontrolled studies, Jamison et al.'s study [5] uniquely addressed aspects of sexuality in Bipolar individuals who were in the euthymic state, conceptualized sexuality in terms of the whole person, highlighted sex differences between men and women in sexual experience, and directed attention to state versus trait sexual phenomena.

Recognizing the value of this approach, we were prompted to investigate diverse aspects of sexuality in relatively stable Bipolar individuals. We also wished to study a group of Bipolar patients underreported in the literature—those treated in routine office practice.

We compared a Bipolar patient group to other psychiatric patients with no affective disorder seen in a private practice setting, with the goal of increasing knowledge about their sexual behavior. We expected Bipolar patients to have more sexual thoughts and behaviors than other patients treated by private practitioners, even when Bipolar patients were euthymic and on medication.

## 2. Study Methods

### 2.1. Method of Recruiting Subjects

One purpose in carrying out this investigation was to utilize a patient population different from that usually reported in scientific studies. Psychiatrists in private practice often obtain valuable information that is not routinely published. We collected such information by systematically querying each psychiatrist in our group about the characteristics of patients that he/she knew well, having treated them for months and even years. The data collected would therefore supplement that usually reported in studies of more conventional design. Participating psychiatrists were all psychodynamically trained and provided both psychotherapy and psychotropic medication to the patients studied. They knew the patients in depth. This was an advantage since subjects may be reticent to report sexual information in questionnaire studies or to impart such information to researchers with whom they have no relationship. Since data were coded and only reported in aggregate, no individual patient could be identified. All study procedures were approved by the New York State Psychiatric Institute Institutional Review Board. Participants gave written informed consent.

Patients (*N* = 76) were psychiatric outpatients of the study psychiatrists, all members of the Human Sexuality Committee of the Group for the Advancement of Psychiatry (GAP), an affiliate organization of the American Psychiatric Association (APA). These patients had presented for treatment with diverse symptoms and complaints. No patient was aware that his or her psychiatrist was a member of a study group on sexual behavior or Bipolar disorder. The patients that each psychiatrist reported on therefore were those that happened to be part of his or her routine office practice (total number of psychiatrists = 7: M = 3 and F = 4).

Psychiatrists had practices in the Far West (1), Rocky Mountain states (1), and Northeastern United States (5). All Bipolar patients and 59 percent of Comparison patients were taking at least one psychotropic medication (see below for details).

### 2.2. Inclusion Criteria

In order to be reported on, patients had to be 18 years of age or older, to have attended at least four appointments in the 12 months before the study, and to have been seen during the last five business days before the study data collection period. Each psychiatrist reported on* every* patient that met inclusion criteria for Bipolar disorder or Comparison group as described below. This means that subject recruitment was* consecutive* for the period of time studied. Patients whose symptoms were in remission were included so long as they were still in treatment.

Psychiatrists filled out a questionnaire for each patient, based on what they knew about him or her (see below). Although they did not further query any patient in order to answer the questions, they consulted patients' charts as needed. We felt it would be intrusive for psychiatrists to suddenly inquire about specific aspects of their sexual behavior.

### 2.3. Method of Information Collection

The study questionnaire, titled* Sexual History Form*, was created by the GAP Committee on Human Sexuality and may be obtained from the senior author (Jennifer Downey at jid1@cumc.columbia.edu). The questionnaire consisted of demographic (Q1–10), psychiatric (Q11–31), and sexual history questions (Q32–72) and took 30–45 minutes to fill out for each patient. Sexual history questions fell into the following categories, all for the last year: Current Sexual History (Q32–35), Nature of Partners (Q36–42), Frequency of Sexual Behaviors (Q43–54), Sexual Risk-taking (Q55–58), STD status (Q59-60), Sexual Orientation Identity and Gender Identity (Q61–64), Hormonal Status for Female Patients (Q65), and Special Circumstances Affecting Sexuality (Q66–69). The last section asked about presence of probable Bipolar disorder in the patient's sexual partner and family of origin (Q70-71) and clarification of any answer (Q73). These last 3 questions did not specify a period of time.

All DSM IV-TR diagnoses were listed for each patient [2]. The Global Assessment of Functioning Scale (GAF) [19] was used to assess average overall psychological function for the past year. A second GAF score was assigned to assess the lowest level of functioning during a one-week period in the last year. Episodes of affective illness in the last year were assessed as were psychotropic medications. Questions concerning lifetime psychiatric illness included age of onset at first illness and number of psychiatric hospitalizations. Diagnosis of Bipolar disorder in the patient's family was also assessed. The remainder of the psychiatric items concerned the nature and severity of the worst episode of mania or hypomania the patient had ever had.

### 2.4. Method of Data Management and Analysis

IBM SPSS was used for data analysis. In preliminary analyses we examined the distribution, means, and standard deviations of the demographic and other continuous measures, as well as the distributions of categorical measures. All but three continuous measures were found to be normally distributed (see below). There was a series of questions regarding specific sexual practices (Q43–51) for which “Don't know” was the most common response. These data are not presented.

Seven questions (Q36–42) address the “Nature of Partners In The Past Year,” ranging from primary partners to strangers to paid phone/internet sex. The affirmative answers to these questions were summed and considered a partner risk measure. Items Q55, 57, and 58 (substance use, sex with strangers, and anal/vaginal intercourse without condoms with nonmonogamous partners) were also summed as a risk-taking measure.

Continuous measures were compared across the two patients groups using the *t*-test statistic. If Levene's test indicated unequal variances (Age in years @ onset 1st Psychiatric Illness, Duration of Current Treatment (months), and Hypomanic/Manic Symptoms), we report the separate variance estimates for the *t*-test and significance level. For the categorical measures, many of which exhibited small cell frequencies, we used Fisher's Exact Test (FET) throughout. All tests were two-tailed and the alpha for significance was set at *p* < 0.050.

## 3. Results

### 3.1. Subject Characteristics

Psychiatric diagnoses are presented first since diagnosis (plus the treatment characteristics noted above) was the criterion by which patients were selected.

### 3.2. Psychiatric Diagnosis of the Subjects

The Bipolar group consisted of individuals diagnosed with a primary diagnosis of one of the Bipolar disorders according to DSM-IV-R criteria. Total *N* was 32. Of these individuals *N* = 10 were receiving treatment for Bipolar I disorder, *N* = 20 were receiving treatment for Bipolar II disorder, and *N* = 2 bore the diagnosis Bipolar disorder NOS.

The Comparison group of psychiatric outpatients consisted of individuals who never had an episode of affective illness, either Bipolar disorder or unipolar depression. Total *N* was 44. Of these, the majority were receiving treatment for the primary diagnosis of an Anxiety Disorder (*N* = 24) or Adjustment Disorder with Anxiety (*N* = 7). Other primary diagnoses were lightly represented: Attention Deficit Disorder *N* = 1, Substance Abuse Disorder *N* = 1, Posttraumatic Stress Disorder *N* = 1, Pain Disorder *N* = 1, and Delusional Disorder *N* = 1. Only one patient had no Axis I diagnosis; that person was being treated for Personality Disorder NOS.

A number of patients in each study group had more than one Axis I disorder. The Bipolar group of *N* = 32 had nine patients with a second Axis I disorder and three patients with a third Axis I disorder. Of the 44 patients in the Comparison group, nine had a second Axis I diagnosis, four had a third Axis I disorder, and two had a fourth Axis I disorder.

Some patients also had Axis II disorders. Of the Bipolar group, 8 patients had received diagnosis of diverse Axis II conditions.

Of the Comparison group, 16 patients had received Axis II diagnoses.

Subject demographics are listed in Table 1. All subjects were at least 18 years of age and were English-speaking. The Bipolar group consisted of 16 males and 16 females. The Comparison group consisted of 26 males and 18 females. Average age for both groups was early 40s. Slightly over than 50% of each subject group had a live-in partner.

Educational level for the patients in both groups was high. The majority of patients were college graduates or had a graduate degree. The Comparison group had a higher occupational status according to the Hollingshead Scale [20] and also a higher calculated Hollingshead Socio-Economic Status, which uses both educational and occupational levels.

*Additional Psychiatric Information about the Subjects*. As noted above, the Global Assessment of Functioning (GAF) scale was used to measure overall psychological functioning. This psychometrically valid and reliable instrument yields scores varying from 1 to 90 with 90 being good functioning in all areas and 1 being functioning so severely impaired that the patient's life is threatened. Although the average GAF for the last year did not differ significantly for the Bipolar and Comparison groups, the Bipolar group differed from the Comparison patient group on a variety of other parameters, demonstrating more impairment in the Bipolar individuals. For instance, GAF score for the lowest-functioning week over the last year was significantly lower for the Bipolar group. Statistical tests and significance levels for all parameters discussed in this section may be found in Table 2.

Other measures of psychopathology also showed the Bipolar group to be generally more severely affected. Thus, mean age of onset of psychiatric symptoms in the Bipolar group was 15.7 years while the Comparison group had onset at 21.3 years. Duration of present treatment differed by more than 30 months on average with Bipolar individuals having been in treatment longer. This was not a significant difference due to large standard deviations. More patients with Bipolar disorder were reported to be taking psychiatric medications than Comparison patients (100% versus 59%; Yates *χ* ^2^ = 14.92, *p* < 0.001) and to have experienced psychiatric hospitalization (FET: *p* < 0.0001).

Patients with Bipolar disorder were more likely to be taking antipsychotics and mood stabilizers. There were no differences between the patient groups in percentages taking SSRI antidepressants, other antidepressants, anxiolytics, stimulants, sleep medications, and phosphodiesterase inhibitors.

Psychiatrists were asked whether the patient's parents, siblings, or children had definite or probable Bipolar disorder. Over 50% of B patients but only 4.5% of C patients had at least one Bipolar relative related by blood, a highly significant difference.

### 3.3. Sexual History: Whole-Group Comparison

The Bipolar patients were more likely to report sexual experiences that may be described as nontraditional than were the Comparison subjects. Although this might not have been revealed in comparisons of individual items because of relatively small numbers of subjects, the* sum* of affirmative items (Q36–42) regarding nature of sexual partners was statistically significant (*p* = 0.05 by *t*-test).

In other ways the groups were similar in sexual behavior over the previous year. For instance, patients in both groups reported an average of “moderate enjoyment” of their sex lives (Q32). Mean frequency of sexual activity with another person over the last year was “sometimes” defined as two to eight times/month (Q34).

Questions 43–51 and 53-54 concerned the frequency of certain sexual behaviors/experiences—masturbation, orgasm, sexual desire, and novelty-seeking sexual behavior—for the year and for the most intense week of the year. For these items there were too much missing data for analyses to be meaningful, and the results are not presented here. This was a result of research design. The practicing clinicians did not always have readily available details about the patients' most intense week in the last year. If the questionnaire had been administered to patients directly, this information would have been straightforward to elicit.

Another type of question assessed diagnosis of a DSM-IV Sexual Dysfunction or Paraphilia (Q52). Very few patients in either group had received one of these diagnoses (Bipolar *N* = 4 versus Comparison *N* = 3), and there was no difference between the groups.

Sexual risk-taking was assessed with six questions (Q55–60). We found no significant differences between Bipolar and Comparison groups for any of the items. The first four were frequency with which they engaged in recreational substance use with sex (Q55), substances they used if any (Q56), sex with strangers (Q57), and receptive anal or vaginal intercourse without a condom with partners of unknown HIV status (Q58). One person in each group was known to have positive HIV status, not a significant difference. There was also no difference in how many patients had been diagnosed with any other STD in the last year.

Sexual orientation identity and gender identity were assessed with four questions (Q61–64). Sexual* activity* in the last year was rated by Kinsey scale where 0 is exclusively heterosexual activity and 6 is exclusively homosexual activity [21]. The two groups did not differ in sexual behavior Kinsey scores. The majority were reported to have engaged exclusively in heterosexual activity in the last year.

A separate question asked was for the psychiatrists to rate patients' sexual* fantasy* over the last year using the same Kinsey scale. Again, the two patient groups did not differ. Thirty-four percent of Bipolar patients and 45.5 percent of Comparison patients were reported to have had only heterosexual fantasies. Thirteen percent of Bipolar and 16 percent of Comparison patients (*N* = 7) had had only homosexual fantasies. A fairly substantial number of patients experienced both heterosexual and homosexual fantasies at times—Bipolar 22 percent versus Comparison 9 percent. Psychiatrists reported not enough information to rate Kinsey fantasy level for about 30 percent of the patients.

Sexual orientation self-identity in the last year (Q63) was assessed separately from the Kinsey scale questions and was found not to differ significantly between groups. Most patients were reported by their psychiatrists as having a “straight” identity. The percentages with a self-identity as gay or lesbian were about the same for both groups—16 percent versus 18 percent (Bipolar versus Comparison). One Bipolar person was reported to have a bisexual identity and one to have “other.” Gender identity in the last year was exclusively male or female in all cases except for the one Bipolar patient reported as transgender, male to female.

Special circumstances which might affect sexual functioning were assessed with five questions (65–69). Hormonal status was assessed for women in both groups and did not differ. Most women were premenopausal.

When the entire Bipolar and Comparison groups, both males and females, were compared, there were no special circumstances over the last year reported that might affect sexual functioning (falling in love, pregnancy, ill health, and sexual abuse) that differed between groups.

A question (70) assessed bipolarity in the patients' relationships. One patient in each group was reported to definitely or probably have a Bipolar partner.

### 3.4. Sexual History: Analysis of Subgroups

Additional information sometimes emerges when statistical tests are performed on subgroups of interest. We looked at two additional comparisons for sexual history items. We compared patients by gender so that Bipolar males were compared to Comparison males, and Bipolar females were compared to Comparison females. We also compared Bipolar males with Bipolar females and Comparison males with Comparison females.

For the comparison between men, more Bipolar patients were reported to have had additional lovers (*p* = 0.047 by Fisher's Exact Test, question 37). There was a trend for Bipolar men to have engaged in receptive intercourse without condoms in the last year relative to the Comparison group (*p* = 0.061 by Fisher's Exact Test; question 58). Comparisons of Bipolar with Comparison female patients showed no differences.

Finally, meaningful differences in the sexual behavior of the men versus women in each group were examined. Bipolar males differed from Bipolar females on two items only. More Bipolar males than females had had two or more sexual partners *n* the last year—males 67% versus females 19% (*p* = 0.034 by Fisher's Exact Test, question 33). More Bipolar males than females had engaged in homosexual activity in the last year (*p* = 0.018 by Fisher's Exact Test, question 61). The easiest way to understand this since the Kinsey score was on a spectrum, not binary, is to consider that 67% of Bipolar males but 94% of Bipolar females had engaged only in heterosexual activity in the last year. There was also a trend for Bipolar males to be more likely than females to have a homosexual identity (*p* = 0.083 by Fisher's Exact Test, question 63).

Comparisons of Comparison males to females showed that Comparison males had engaged in more frequent sexual activity during the most intense week over the last year (*p* = 0.049 by Fisher's Exact Test, question 35). More males than females were reported to have engaged in the last year in homosexual activity (*p* = 0.012 by Fisher's Exact Test, question 61) and in homosexual fantasy (*p* = 0.037 by Fisher's Exact Test, question 62). When sexual orientation self-identity was reported, eight Comparison men but no women reported a gay/lesbian identity (*p* = 0.014 by Fisher's Exact Test, question 63).

### 3.5. Sexual History: Risk-Taking among All Patients

Another way to look at the sexual risk-taking among this outpatient sample is to note how many people had engaged in these behaviors* ever* in the last year. Despite the fact that these patients had an average age in their 40s, were married, were highly educated, and were in an ongoing therapy with a psychiatrist, a surprising number of patients in both groups had engaged in risky sexual behavior over the last year. For instance, the number of patients who engaged in recreational drug use and sex at the same time (Q55) was over 40 percent for each group—Bipolar = 46.9 percent (*N* = 15) versus Comparison = 43.2 percent (*N* = 19). The percentage of patients who had engaged in sex with a stranger over the last year (Q57) was over 20 percent—Bipolar = 26 percent (*N* = 8) versus Comparison = 21 percent (*N* = 8). The number of patients who had engaged in receptive anal and/or vaginal intercourse without condoms with partners not known to be monogamous was 19% (*N* = 6) for the Bipolar individuals and 8% (*N* = 3) for the Comparison patients.

## 4. Discussion

This naturalistic comparison study of the sexual experience and activity of Bipolar patients had unique design features. We hoped that our research approach might demonstrate the usefulness of systematic investigation of patients not usually studied and of harvesting data that is often not shared in the professional community. In our view the method we devised could be adapted for the investigation of diverse types of patients other than those with Bipolar disorders and of diverse forms of behavior other than sexuality.

To the best of our knowledge there has been no previous controlled study of sexuality in Bipolar outpatients carried out in such depth and detail. Indeed studies of sexuality in psychiatric patients have been scant. In the case of our own study, the patients received both psychotherapy and medication from their doctors and had largely durable and positive working alliances with them. This is clear from the mean duration of treatment with the psychiatrists—almost four years for the Comparison group and over six years for the Bipolar group. To learn about sexuality, the topic of the questionnaire discussed in this report, the trusting therapeutic relationships that our respondents had with these patients proved crucial.

### 4.1. The Questionnaire

The psychiatrists who provided study data were all members of the Human Sexuality Committee of Group for the Advancement of Psychiatry. Based on an inclusive review of the literature in Bipolar disorder, the group had constructed a questionnaire, which they subsequently filled out about the patients. Respondents had no hypothesis about which specific aspects of sexual experience and activity would be increased.

### 4.2. Validity of Diagnosis of Bipolar Disorder

A crucial question about the sample reported on in this study is whether their psychiatric disorders were accurately diagnosed. The usual indices of diagnostic accuracy are reliability and validity. No effort was made to establish diagnostic reliability in our study using commonly accepted methods. Psychiatrist respondents were experienced in using the DSM-IV-TR and carefully reviewed diagnostic criteria for each subject selected.

A number of measures indicate that the diagnosis of the Bipolar patients was* valid*. For example, among Bipolar patients, more than half had first-degree relatives with definite or probable Bipolar disorder. Only two of the Comparison individuals had a first-degree relative with Bipolar disorder. The difference between the groups was highly significant. This is meaningful because of Bipolar disorder clusters among family members [1].

Given that individuals with schizophrenia and mood disorders were excluded from the Comparison group, it is not surprising that the Bipolar group had had more episodes of severe illness during the course of their lives than the Comparison group. Over one third of Bipolar patients (*N* = 12) had been psychiatrically hospitalized in contrast to only one Comparison patient.

Although the current GAF score between the two groups was similar, those in the Bipolar group were likely to have been more impaired during the worst week of the past year (mean score of 54.7 or “serious impairment”), while mean score for Comparison patients during the worst week was 64.8 or “moderate symptoms.”

Mean age of onset of psychiatric symptoms in the Bipolar group was significantly younger than in the Comparison group (15.6 versus 21.2 years), compatible with the overall trend of psychopathology in the sample.

In keeping with what has been reported elsewhere [22], Bipolar patients in this study took more than twice as many psychiatric medications as Comparison patients and were much more likely to be prescribed antipsychotic medicines. Perhaps more importantly as another indicator of validity, 71.9 percent of Bipolar patients were prescribed thymoleptic mood stabilizers, in contrast with no Comparison patients. This finding is both statistically and clinically significant.

The fact that the Bipolar patients and Comparison patients were well-balanced with respect to use of SSRI medications is particularly important given the well-known sexual inhibitory effects of these drugs [23]. Of course, the SSRI drugs might also induce hypomanic or manic states in predisposed patients, but that did not appear to occur in the population we report.

It is important for diagnostic accuracy that no patient with unipolar depression was included in the Comparison group. In two surveys of members of the National Depressive and Mani-Depressive Association (DMDA), most Bipolar patients were initially misdiagnosed [24, 25]. The most common incorrect diagnosis was unipolar depression. This may have been because the first affective episode of Bipolar disorder is often depression.

### 4.3. Comorbidity

Of the Bipolar patients in our study, nine (28 percent) had more than one Axis I disorder and eight (25 percent) had Axis II disorders. The Comparison patients were similar in this regard, with nine (20 percent) having more than one Axis I disorder and 16 (36 percent) having an Axis II disorder.

These findings are consistent with what might be expected from published studies showing significant comorbidity in Bipolar individuals [26, 27]. Comorbidity prevalence with Bipolar disorder varies substantially across studies and may be as high as 75 percent [1].

### 4.4. Sexuality Findings

Our group of psychiatrists had discussed the literature on Bipolar disorder and sexuality among ourselves and expected our Bipolar patients to be more sexual in some sense than controls. This expectation was only modestly supported by the pooled data, however.

The group of Bipolar patients had more sexual partners than did Comparison patients and increased frequency of risky sexual behavior.

In addition, Bipolar males had more sexual partners during the past year than Bipolar females and more Bipolar males had engaged in homosexual activity. Bipolar males were also somewhat more likely than Comparison males to have engaged in receptive intercourse without condoms during the past year.

### 4.5. Similarity in Sexual Behavior between Bipolar and Comparison Patients

We note that risky sexual behavior has been reported to be increased in psychiatric patients generally [28, 29]. Some studies have reported risky behavior among psychiatric patients to be increased in females compared to males [30]. A recent study reported high-risk behavior to be increased during hypomanic states, generally [31].

Our investigation did find certain risky behaviors to be increased particularly in the males who were Bipolar, but these findings were modest. One reason for this may well have been that every patient with Bipolar disorder in this investigation was medicated. Although the patients were treated with psychotherapy as well, we conjecture that the dimensions of sexual motivation that were embedded in hypomanic or manic affective tendencies were diminished by pharmacological agents. It might also be that the particular patients reported on in this sample, although clearly Bipolar, were not drawn from the most extreme segment of the illness spectrum, particularly at the time of data collection.

An astonishing and clinically important finding of this investigation was the frequency of unconventional and risky sexual behavior among the Comparison patients. This included sex with strangers, failure to use condoms with partners not known to be monogamous, and sex with prostitutes. This indicates the importance of the sexual history in the general assessment of psychiatric patients, not just Bipolar individuals.

## Figures and Tables

**Table 1:** Demographics: statistics are the *t*-test for continuous measures and the Fisher's Exact Test for categorical measures.

Demographic measures	Bipolar disorders		Comparison				
	*N* = 32		*N* = 44		Group effect		
	Mean	sd	Mean	sd	*t*	df	*p*
Age	41.6	(13.2)	42.2	(14.3)	0.17	74	0.869
Hollingshead occupation code	7.4	(1.5)	8.1	(1.2)	2.10	67	0.040^*∗*^
Hollingshead SES	55.6	(8.6)	60.1	(6.7)	2.43	67	0.018^*∗*^

	%	*N*	%	*N*	Fisher's Exact *p*		

Gender (female % and *N*)	50	(16)	41	(18)	0.488		
Education level							
High school/GED	3	(1)	0	(0)			
Some college	9	(3)	7	(3)			
B.A./B.S. degree	47	(15)	36	(16)			
Graduate school	41	(13)	57	(25)	0.364		
Current employment status							
Retired	9	(3)	5	(2)	0.644		
Homemaker	16	(5)	2	(1)	0.077^t^		
Unemployed	16	(5)	9	(4)	0.480		
Student	9	(3)	14	(6)	0.726		
Race							
White	88	(28)	96	(42)			
Black	3	(1)	0	(0)			
Asian/PAC. ISL	3	(5)	5	(2)			
Other	6	(2)	0	(0)	0.215		
Hispanic	13	(4)	2	(1)	0.233		
Marital status							
Married	38	(12)	48	(21)			
Single	53	(17)	46	(20)			
Widowed/divorced	9	(3)	7	(3)	0.678		
Live-in partner	56	(18)	57	(25)	1.0		
Religion							
Protestant	28	(9)	30	(13)			
Catholic	19	(6)	14	(6)			
Jewish	28	(9)	43	(19)			
Other	6	(2)	7	(3)			
None	19	(6)	7	(3)	0.460		

^*∗*^indicates significance and ^t^trend.

**Table 2:** Psychiatric history and clinical characteristics.

Clinical measures	Bipolar disorders		Comparison				
	*N* = 32		*N* = 44		Group effect		
	Mean	sd	Mean	sd	*t*	df	*p*
Age in years @ onset 1st Psychiatric Illness	15.7	(5.8)	21.3	(11.7)	2.72	67^†^	0.008^*∗*^
Global assessment of function							
Average over the past year	68.7	(8.7)	72.6	(9.6)	1.80	74	0.076^t^
Lowest week during last year	54.8	(13.7)	64.9	(12.1)	3.41	74	0.001^*∗*^
Duration of current treatment (months)	77.3	(107.1)	46.6	(67.4)	1.43	74^†^	0.158
Hypomanic and manic symptoms	5.7	(1.5)	0.14	(.50)	18.16	46^†^	<0.001^*∗*^

	%	*N*	%	*N*	Fisher's Exact *p*		

Episodes during the past year							
Manic episodes	6	(2)	0	(0)	0.174		
Depressive episodes	31	(10)	0	(0)	0.000^*∗*^		
Mixed episodes	50	(16)	0	(0)	0.000^*∗*^		
Hospitalizations (lifetime)							
0	63	(20)	98	(41)			
1	19	(6)	2	(1)			
2 or more	19	(6)	0	(0)	<0.0001^*∗*^		
Current psychotropic medications							
Antipsychotics	44	(14)	7	(3)	<0.001^*∗*^		
SSRI	47	(14)	32	(14)	0.228		
Antidepressants	23	(7)	14	(6)	0.363		
Mood stabilizers	72	(23)	0	(0)	<0.001^*∗*^		
Anxiolytics	48	(15)	41	(18)	0.638		
Stimulants	13	(4)	9	(4)	0.711		
Sleep	19	(6)	18	(8)	1.00		
Phosphodiesterase	10	(3)	2	(1)	0.300		
Other	16	(5)	5	(2)	0.129		
Number of medications prescribed Mean (sd) *t*-test	2.8	(1.3)	1.3	(1.3)	5.20	74	<0.001^*∗*^
Bipolar family history First degree relatives	53	(16)	5	(2)	<0.001^*∗*^		
Significant medical illness (during the last year)	34	(11)	30	(13)	0.803		

^*∗*^indicates significance, ^†^separate variance est., and ^t^trend.

## Additional Points

Our study had a number of limitations. The convenience group of patients that the therapists reported on was not representative of Bipolar patients generally. The fact that the patients were largely Caucasian and well-educated and able to pay for private treatment limited generalizability of our findings. The greatest strengths of our design were also its greatest weaknesses. Therapists with preconceived beliefs about the questions being studied provided data about their patients. Obviously this might have biased responses. Interestingly, however, the therapists' expectations were frequently not confirmed. The advantage of using the therapeutic alliance to study sexuality seemed apparent to our group. In order to protect the alliance, closed ended questions about sexuality were not part of our design. Therapists provided only historical data that they had acquired from their psychotherapeutic work with patients. We judged that the truthfulness of patient responses, however, outweighed the limitations of this approach. Due to practical limitations we focused only on the past year. The sexual history of patients prior to that was not explored. The longer arc of sexuality in Bipolar individuals over the life cycle remains for others to explore. Another limitation is that we did not use independent standardized measures to document possible temperamental differences between the two groups and to provide additional mood data. This would be straightforward to do in a future study. Similarly, it would be straightforward to administer the questionnaire directly to Bipolar and control individuals in a research clinic, thus obtaining much more information about the fine details of sexual variations (e.g., during the most sexual week) over the time period studied. In conclusion we stress that our study might be thought of at two levels: as research about sexuality in Bipolar patients and as a feasibility study of carrying out research in a private practice setting.

## References

[1] Goodwin F. K., Jamieson K. R. (2007). Sexual Behavior in Manic Depressive Illness. Chapter 2: clinical description and diagnosis, Chapter 10: personality, personality disorders, and interpersonal functioning. *Manic Depressive Illness*.

[2] American Psychiatric Association (2000). *Diagnostic and Statistical Manual of Mental Disorders (DSM-4 TR)*.

[3] American Psychiatric Association (2013). *Diagnostic and Statistical Manual of Mental Disorders (DSM-5)*.

[4] Clayton P. J., Pitts F. N., Winokur G. (1965). Affective disorder. IV: Mania. *Comprehensive Psychiatry*.

[5] Jamison K. R., Gerner R. H., Hammen C., Padesky C. (1980). Clouds and silver linings: positive experiences associated with primary affective disorders. *American Journal of Psychiatry*.

[6] Raja M., Azzoni A. (2003). Sexual behavior and sexual problems among patients with severe chronic psychoses. *European Psychiatry*.

[7] Dell'Osso L., Carmassi C., Carlini M. (2009). Sexual dysfunctions and suicidality in patients with bipolar disorder and unipolar depression. *Journal of Sexual Medicine*.

[8] Mazza M., Harnic D., Catalano V. (2011). Sexual behavior in women with bipolar disorder. *Journal of Affective Disorders*.

[9] Beigel A., Murphy D. L., Bunney W. E. (1971). The manic-state rating scale: scale concentration, reliability and validity. *Archives of General Psychiatry*.

[10] Spalt L. (1975). Sexual behavior and affective disorders. *Disease of the Nervous System*.

[11] Casper R. C., Redmond D. E., Katz M. M., Schaffer C. B., Davis J. M., Koslow S. H. (1985). Somatic symptoms in primary affective disorder. *Archives of General Psychiatry*.

[12] Raboch J. (1986). Sexual development and life of psychiatric female patients. *Archives of Sexual Behavior*.

[13] Frank E., Targum S. D., Gershon E. S. (1981). A comparison of nonpatient and bipolar patient-well spouse couples. *American Journal of Psychiatry*.

[14] Allison J. B., Wilson W. P. (1960). Sexual behavior of manic patients: a preliminary report. *Southern Medical Journal*.

[15] Winokur G., Clayton P. J., Reich T. (1969). *Manic Depressive Illness*.

[16] Carlson G. A., Goodwin F. K. (1973). The stages of mania: a longitudinal analysis of the manic episode. *Archives of General Psychiatry*.

[17] Loudon J. B., Blackburn I. M., Ashworth C. M. (1977). A study of the symptomatology and course of manic illness using a new scale. *Psychological Medicine*.

[18] Akiskal H. S. (2005). Searching for behavioral indicators of bipolar II in patients presenting with major depressive episodes: the ‘red sign,’ the ‘rule of three’ and other biographic signs of temperamental extravagance, activation and hypomania. *Journal of Affective Disorders*.

[19] Endicott J., Spitzer R. L., Fleiss J. L., Cohen J. (1976). The global assessment scale. A procedure for measuring overall severity of psychiatric disturbance. *Archives of General Psychiatry*.

[20] Hollingshead A. B. (1975). *Four Factor Index of Social Status*.

[21] Kinsey A. C., Pomoroy W., Martin C. (1953). *Sexual Behavior in the Human Female*.

[22] Baldessarini R. J., Leahy L., Arcona S., Gause D., Zhang W., Hennen J. (2007). Patterns of psychotropic drug prescription for U.S. patients with diagnoses of bipolar disorders. *Psychiatric Services*.

[23] Corona G., Ricca V., Bandini E. (2009). Selective serotonin reuptake inhibitor-induced sexual dysfunction. *Journal of Sexual Medicine*.

[24] Lish J. D., Dime-Meenan S., Whybrow P. C., Price R. A., Hirschfeld R. M. A. (1994). The National Depressive and Manic-depressive Association (DMDA) survey of bipolar members. *Journal of Affective Disorders*.

[25] Hirschfeld R. M. A., Lewis L., Vornik L. A. (2003). Perceptions and impact of bipolar disorder: how far have we really come? Results of the National Depressive and Manic-Depressive Association 2000 Survey of individuals with bipolar disorder. *Journal of Clinical Psychiatry*.

[26] George E. L., Miklowitz D. J., Richards J. A., Simoneau T. L., Taylor D. O. (2003). The comorbidity of bipolar disorder and axis II personality disorders: prevalence and clinical correlates. *Bipolar Disorders*.

[27] Altindag A., Yanik M., Nebioglu M. (2006). The comorbidity of anxiety disorders in bipolar I patients: prevalence and clinical correlates. *Israel Journal of Psychiatry and Related Sciences*.

[28] Carey M. P., Carey K. B., Maisto S. A., Schroder K. E. E., Vanable P. A., Gordon C. M. (2004). HIV risk behavior among psychiatric outpatients: association with psychiatric disorder, substance use disorder, and gender. *Journal of Nervous and Mental Disease*.

[29] Otto-Salaj L. L., Stevenson L. Y. (2001). Influence of psychiatric diagnoses and symptoms on HIV risk behavior in adults with serious mental illness. *The AIDS Reader*.

[30] McKinnon K., Cournos F., Herman R. (2001). A lifetime alcohol or other drug use disorder and specific psychiatric symptoms predict sexual risk for HIV infection among people with severe mental illness. *AIDS and Behavior*.

[31] Fletcher K., Parker G., Paterson A., Synnott H. (2013). High-risk behaviour in hypomanic states. *Journal of Affective Disorders*.

